# Prevalencia de estomatitis aftosa recurrente en un consultorio médico de familia, Manzanillo, Cuba. Un estudio transversal

**DOI:** 10.21142/2523-2754-1104-2023-172

**Published:** 2023-12-28

**Authors:** Viviana de la Caridad Collado Pérez, Milagros de la Caridad Pérez Suárez, Carlos Manuel Collado Hernández, Vivian Pérez Núñez

**Affiliations:** 1 Hospital Provincial Clínico Quirúrgico Docente “Celia Sánchez Manduley”. Manzanillo, Granma, Cuba. vivicollado1997@gmail.com, rfbanqueris@gmail.com, vivicollado2013@gmail.com Hospital Provincial Clínico Quirúrgico Docente “Celia Sánchez Manduley” Manzanillo, Granma Cuba vivicollado1997@gmail.com rfbanqueris@gmail.com vivicollado2013@gmail.com; 2 Hospital Psiquiátrico Provincial Docente “Manuel Fajardo Rivero”. Manzanillo, Granma, Cuba. vperezn2021@gmail.com Hospital Psiquiátrico Provincial Docente “Manuel Fajardo Rivero” Manzanillo, Granma Cuba vperezn2021@gmail.com

**Keywords:** estomatitis aftosa, prevalencia, factores de riesgo, aphthous stomatitis, prevalence, risk factor

## Abstract

**Introducción:**

La estomatitis aftosa recurrente, también conocida como úlceras aftosas o simplemente aftas, se considera la más común de las lesiones de la mucosa oral.

**Objetivo:**

Describir la prevalencia de la estomatitis aftosa recurrente.

**Método:**

Estudio descriptivo, transversal y prospectivo. Se evaluó a 847 pacientes que acudieron al Consultorio Médico de Familia N.^o^ 28, Comunidad San Francisco, Manzanillo (Cuba), desde el 1 de julio de 2021 hasta el 30 de junio de 2022. Un investigador auxiliar, calibrado y capacitado, evaluó las siguientes variables: clasificación clínica de la estomatitis aftosa recurrente (aftosis menor, aftosis mayor, oaftosis herpetiforme), intensidad del dolor de la lesión, localización de la lesión, factores de riesgo (infección viral, infección bacteriana, alteraciones inmunológicas, alteraciones psicosomáticas, traumas bucales, alteraciones gastrointestinales, factores endocrinos, cuadros alérgicos, herencia, deficiencias hemáticas y nutricionales, tabaquismo), grupo etario, sexo, raza y tiempo de permanencia de la lesión.

**Resultados:**

La estomatitis aftosa recurrente presentó un 30,46%, con mayor frecuencia del grupo de edades 30-39 años (24,42%). La aftosis menor constituyó la de mayor frecuencia, con un 91,09%. El tiempo de permanencia de la lesión promedia 10 a 12 días (37,60%), la localización más frecuente corresponde a la zona de borde y punta de la lengua (32,94%) y la intensidad del dolor más representativa fue la leve (63,18%). La mayor frecuencia entre los factores de riesgo correspondió a las alteraciones psicosomáticas (100%).

**Conclusiones:**

La estomatitis aftosa recurrente tuvo una prevalencia mayor al 30%, con predominio del sexo femenino y adultos jóvenes. La aftosis menor y el tiempo de permanencia mayor de 10 días fueron las más frecuentes. La localización más común estuvo en la lengua y el fondo del surco vestibular, con posible existencia de una relación entre las partes móviles de la boca. El estrés es el principal factor de riesgo, agudizado por la COVID-19.

## Abstract (en)

**Introduction:**

Recurrent Aphthous Stomatitis, also known as aphthous ulcers or simply aphthous, is considered the most common of oral mucosal lesions.

**Objective:**

To describe the prevalence of recurrent aphthous stomatitis.

**Methods:**

Descriptive, cross-sectional and prospective study. 847 patients who attended the Family Medical Office No. 28, San Francisco comunity, Manzanillo, from July 1, 2021 to June 30, 2022, Cuba, were evaluated. A calibrated and trained assistant investigator evaluated the following variables: Clinical classification of recurrent aphthous stomatitis (minor aphthosis, major aphthosis, or aphthosis herpetiformis), lesion pain intensity, lesion location, and risk factors (viral infection), bacterial infection, immunological alterations, psychosomatic alterations, oral trauma, gastrointestinal alterations, endocrine factors, allergic conditions, heredity, blood and nutritional deficiencies, smoking), age group, sex, race, and duration of the lesion.

**Results:**

Aphthous stomatitis occurred in 30.46%, with greater frequency in the age group 30 - 39 years (24.42%). Minor aphthosis was the most frequent with 91.09%. The duration of the lesion of 10 to 12 days predominated with 37.60%, the most frequent location corresponded to the edge and tip of the tongue with 32.94% and the most representative pain intensity was mild with a total of 63.18%. The highest frequency among the risk factors corresponded to psychosomatic alterations with 100%.

**Conclusions:**

Recurrent Aphthous Stomatitis had a prevalence greater than 30% with a predominance of the female sex and young adults. Minor Aphtosis and a stay time of more than 10 days were the most frequent. The most common location is the tongue and bottom of the vestibular sulcus with the possible existence of a relationship between the mobile parts of the mouth. Stress, the main risk factor, exacerbated by Covid-19.

## INTRODUCCIÓN

Las enfermedades de la cavidad bucal están catalogadas entre las afecciones más comunes del género humano ^1^. Entre ellas podemos mencionar algunas que ocasionan signos y síntomas que pueden preocupar a los pacientes: caries dental, pulpitis aguda serosa transitoria, conductos radiculares infectados, alveolitis, pericoronaritis, gingivitis crónica edematosa, estomatitis subprotésica y estomatitis aftosa recurrente (EAR), entre otras ^2^.

Una de las enfermedades periodontales más representativas por su frecuencia y sintomatología es la estomatitis aftosa recurrente, por ser la más común entre las enfermedades de la mucosa bucal ^3^.

Esta afección inflamatoria aguda está catalogada como una de las urgencias estomatológicas más frecuentes ^(4, 5)^ y puede requerir atención inmediata por urgencia debido a las molestias que ocasiona a quien la padece ^6^.

La estomatitis aftosa recurrente -también conocida como úlceras aftosas o, simplemente, aftas- se considera la más común de las lesiones de la mucosa oral, pues afecta alrededor del 20% de la población general, principalmente adolescentes y adultos jóvenes ^7^.

Es una lesión que se inicia con vesículas esféricas circunscritas que se rompen después de un día o dos, y forman úlceras dolorosas, comúnmente nombradas aftas ^8^. Entre las urgencias periodontales que requieren atención inmediata está la estomatitis aftosa recurrente, debido a las molestias o complicaciones que ocasiona al paciente ^9^.

Desde hace muchos años, las úlceras aftosas han significado un problema cotidiano y muy común dentro de las patologías bucales. Sin embargo, tal vez sean las que han merecido una menor importancia, debido a su remisión (tienen una duración de 7 a 10 días) y la inexistencia de un tratamiento eficaz y específico. Si se reducen los días en la curación, disminuyen los riesgos de complicaciones en estas úlceras orales, ya que constituyen una puerta de entrada para las infecciones oportunistas ^10^.

La alta frecuencia, la distribución mundial, el amplio rango de edad, el carácter recurrente de la enfermedad y la disminución de la calidad de vida de los pacientes con EAR han conducido a una gran cantidad de investigaciones sobre la etiología y el tratamiento de esta afección ^10^.

En países como Irán (2005), Jordania (2008), India (2010-2012) ^11^ y China (2013-2017) ^12^ se reporta una prevalencia del 25,2%, 70%, 21,7% y 27,17%, respectivamente ^11^^,^^12^.

Un estudio brasileño realizado en adolescentes masculinos encontró un 24,9 % de prevalencia de EAR ^13^. Otra investigación brasileña en 4895 pacientes atendidos en clínica odontológica presentaron 3,3% úlceras aftosas orales, de los cuales el 47,2% sufrían de EAR ^14^.

En la provincia de Granma se han publicado trabajos investigativos sobre la estomatitis aftosa que evalúan diferentes características clínico-epidemiológicas, específicamente en la consulta de periodoncia de la Clínica de Especialidades Estomatológicas, en el municipio de Bayamo ^15^.

El aumento de los pacientes que acuden a la consulta o cuerpo de guardia con aftas bucales en nuestra área de salud motivó a emprender este proceso investigativo, que permitió abordar aspectos clínicos epidemiológicos de la enfermedad, como el comportamiento y la frecuencia para conocer y dominar mejor sus características, a fin de prevenir su aparición, así como mejorar la salud bucal de nuestros pacientes. Por lo tanto, el propósito de esta investigación fue describir la prevalencia de la estomatitis aftosa recurrente en el Consultorio Médico de la Familia en Manzanillo, Cuba.

## MATERIALES Y MÉTODOS

El estudio fue realizado teniendo en cuenta lo establecido en la declaración de Helsinki sobre la investigación en seres humanos. Se cumplieron las normas éticas en cuanto a la discreción, confiabilidad de la información y honestidad. Fue aprobado por el comité de ética del Hospital Provincial Clínico Quirúrgico Docente Celia Sánchez Manduley, con el número 174/2022.

Este estudio descriptivo, transversal y prospectivo fue realizado en el Consultorio Médico de la Familia N.^o^ 28 perteneciente a la comunidad de San Francisco, municipio de Manzanillo, provincia de Granma, desde el 1 de julio de 2021 hasta el 30 de junio de 2022, con el objetivo de describir la prevalencia de la estomatitis aftosa recurrente en la población de esta comunidad.

Un evaluador investigador auxiliar, calibrado y capacitado, evaluó las siguientes variables: grupos etario, sexo (femenino y masculino), raza (blanca, mestiza, negra), tiempo de permanencia de la lesión (1 a 3 días, 4 a 6 días, 7 a 9 días, 10 a 12 días, 13 a 15 días), clasificación clínica de la estomatitis aftosa recurrente (aftosis menor, aftosis mayor, aftosis herpetiforme), intensidad del dolor de la lesión (leve, moderado, severo), localización de la lesión (mucosa interna de los labios, suelo de boca, fondo de surco vestibular, bordes y punta de la lengua, mucosa yugal -carrillos-, paladar, encías) y factores de riesgo (infección viral, infección bacteriana, alteraciones inmunológicas, alteraciones psicosomáticas, traumas bucales, alteraciones gastrointestinales, factores endocrinos, cuadros alérgicos, herencia, deficiencias hemáticas y nutricionales, tabaquismo).

La información necesaria para la presente investigación fue recopilada en un modelo confeccionado para ello. Esta información se obtuvo a partir de la anamnesis y el examen de la cavidad bucal que se le realizó a cada paciente.

### Plan de análisis

Para el procesamiento de la información se utilizó una computadora marca Asus con sistema operativo Windows 10 y *software* de procesamiento estadístico Microsoft Excel para Windows 10. Los resultados se expusieron en números absolutos y porcentajes que se expresaron en tablas.

## RESULTADOS

Del total de 847 pacientes que forman los grupos de edades estudiados y que se muestran en la Tabla 1, 258 (30,46%) presentaron estomatitis aftosa recurrente. Cuando se realiza un análisis de los casos según el sexo, observamos un predominio del femenino con 141 casos (16,65%).

**Tabla 1:** Distribución de estomatitis aftosa recurrente según población general de 10 a 69 años y sexo

Sexo	Pacientes	%	Pacientes con EAR	%
Femenino	467	55,14	141	16,65
Masculino	380	44,86	117	13,81
Total	847	100	258	30,46

En la Tabla 2 encontramos una mayor frecuencia de EAR dentro del grupo de edades de 30-39 años con 63 casos (24,42%), seguidos por el grupo de 20-29 años con 50 (19,38%) y 40-49 años con 49 (18,99%). Al analizar los resultados, se observa una mayor frecuencia en el sexo femenino (dentro de los pacientes con EAR), representados con 141 pacientes (54,65%).

**Tabla 2:** Distribución de estomatitis aftosa recurrente según grupos de edades y sexo

Grupo de Edades	Sexo					
	Femenino		Masculino		Total	
	n	%	n	%	n	%
10 a 19 años	21	8,14	15	5,82	36	13,96
20 a 29 años	31	12,02	19	7,36	50	19,38
30 a 39 años	34	13,18	29	11,24	63	24,42
40 a 49 años	25	9,69	24	9,30	49	18,99
50 a 59 años	19	7,36	21	8,14	40	15,50
60 a 69 años	11	4,26	9	3,49	20	7,75
Total	141	54,65	117	45,35	258	100

El tiempo de permanencia de la lesión con respecto a la clasificación clínica de la estomatitis aftosa recurrente se presenta en la Tabla 3, donde la aftosis menor constituyó la de mayor frecuencia con 235 pacientes (91,09%). Cuando se analizó el tiempo de permanencia de la lesión, la de mayor frecuencia fue de 10 a 12 días con 97 casos (37,60%). Le siguen en orden de frecuencia de 13 a 15 días, con 91 casos (35,27%), y de 7 a 9 días, con 65 casos (25,19%).

**Tabla 3:** Distribución de estomatitis aftosa recurrente según tiempo de permanencia de la lesión y clasificación clínica

Tiempo de permanencia de la lesión	Clasificación clínica de la estomatitis aftosa recurrente							
	Aftosis menor		Aftosis mayor		Aftosis herpetiforme		Total	
	n	%	n	%	n	%	n	%
1-3 días	1	0,39	0	0	0	0	1	0,39
4-6 días	4	1,55	0	0	0	0	4	1,55
7-9 días	64	24,80	0	0	1	0,39	65	25,19
10-12 días	89	34,50	7	2,71	1	0,39	97	37,60
13-15 días	77	29,85	12	4,65	2	0,77	91	35,27
Total	235	91,09	19	7,36	4	1,55	258	100

De igual manera en los casos de aftosis menor como más frecuente dentro de la clasificación, predominaron estos periodos de tiempo de permanencia de la lesión: 10 a 12 días, con 89 casos (34,50%); 13 a 15 días, con 77 casos (29,85%), y de 7 a 9 días, con 64 casos (24,80%).

En la Tabla 4 se presenta la localización de las lesiones y la intensidad del dolor. La localización más frecuente correspondió a la zona de borde y punta de la lengua, con 85 casos (32,94 %), seguida por el fondo del surco vestibular, con 61 casos (23,64%), y el suelo de la boca, con 40 casos (15,50%).

**Tabla 4:** Distribución de estomatitis aftosa recurrente según localización de la lesión e intensidad del dolor

Localización de la lesión	Intensidad del dolor							
	Leve		Moderado		Severo		Total	
	n	%	n	%	n	%	n	%
Mucosa interna de los labios	18	6,98	8	3,10	0	0	26	10,08
Suelo de la boca	22	8,52	17	6,59	1	0,39	40	15,50
Fondo de surco vestibular	35	13,57	24	9,30	2	0,77	61	23,64
Bordes y punta de la lengua	52	20,15	31	12,02	2	0,77	85	32,94
Mucosa yugal (carrillos)	16	6,20	4	1,55	1	0,39	21	8,14
Paladar	11	4,27	4	1,55	0	0	15	5,82
Encías	9	3,49	1	0,39	0	0	10	3,88
Total	163	63,18	89	34,50	6	2,32	258	100

Con relación a la intensidad del dolor, el más representativo fue el dolor leve, con un total de 163 pacientes (63,18%). Cuando analizamos a los pacientes que presentaron dolor leve por las lesiones de la estomatitis aftosa recurrente, encontramos que existió de igual manera un predominio en la zona del borde y punta de la lengua, con 52 casos (20,15%); el fondo del surco vestibular, con 35 casos (13,57%), y el suelo de la boca, con 22 casos (8,52%).

La identificación de los factores de riesgo relacionados con el sexo se muestra en la Tabla 5. Es evidente que la mayor frecuencia entre los factores de riesgo correspondió a las alteraciones psicosomáticas, con 258 pacientes (100%), seguidas por las alteraciones gastrointestinales, con 84 casos (32,56%).

**Tabla 5:** Distribución de estomatitis aftosa recurrente según factores de riesgo y sexo

Factores de riesgo (n = 258)	Sexo					
	Femenino		Masculino		Total	
	n	%	n	%	n	%
Infección viral	3	1,16	1	0,39	4	1,55
Infección bacteriana	2	0,77	1	0,39	3	1,16
Alteraciones inmunológicas	3	1,16	1	0,39	4	1,55
Alteraciones psicosomáticas	141	54,65	117	45,35	258	100
Traumas bucales	4	1,55	8	3,10	12	4,65
Alteraciones gastrointestinales	51	19,77	33	12,79	84	32,56
Factores endocrinos	4	1,55	3	1,16	7	2,71
Cuadros alérgicos	8	3,10	6	2,33	14	5,43
Herencia	7	2,71	5	1,94	12	4,65
Deficiencias hemáticas y nutricionales	9	3,49	5	1,94	14	5,43
Tabaquismo	3	1,16	9	3,49	12	4,65

Al analizar la relación en ambos sexos, predominaron iguales factores de riesgo. En el femenino, las alteraciones psicosomáticas, con 141 casos (54,65%), y las alteraciones gastrointestinales, con 51 casos (19,77%). En el masculino prevalecieron las alteraciones psicosomáticas, con 117 (45,35%), y las alteraciones gastrointestinales, con 33 (12,79%).

## DISCUSIÓN

La estomatitis aftosa recurrente es una enfermedad multifactorial frecuente como urgencia en las consultas de estomatología ^4^. En este estudio se encontró que más del 30% de la población estudiada presentó esta enfermedad.

Estos resultados coinciden con los de Queiroz ^16^, en Brasil, donde la prevalencia de la EAR se presenta entre un 5% y un 60%, lo que coincide con los resultados de este trabajo. Por otra parte, otros trabajos realizados en Cuba obtuvieron resultados inferiores, como el realizado por Díaz Couso ^1^, que muestra menos del 10% de la población estudiada. La prevalencia mayor en nuestro estudio puede estar dada por la situación epidemiológica de estrés sobreañadido por causa de la epidemia de COVID-19.

La presencia de EAR en aproximadamente 1 de cada 3 personas de la población estudiada puede estar relacionada con el alto índice de esta enfermedad entre la población, tanto en Cuba como en el resto del mundo, influenciada por diversos factores presentes en la actualidad entre la población en general. De igual manera, el predominio del sexo femenino coincide con investigaciones realizadas con diversos autores a nivel internacional ^17^^,^^18^. En el país evaluado (Cuba), igualmente se encuentra que la mayor frecuencia en varios estudios da al sexo femenino, lo que coincide con los resultados encontrados en esta investigación ^2^^,^^4^^,^^19^. Estos resultados pueden deberse a la influencia de las características fisiológicas del sexo femenino que llevan al desencadenamiento de esta enfermedad ^19^.

La mayor frecuencia fue encontrada en los adultos jóvenes y disminuyó a medida que aumentaba la edad, resultado que coincide con los de investigaciones realizadas en la India ^20^ y Chile ^11^. En Cuba ocurre lo mismo, como lo demuestra Tamayo^4^ en su investigación, donde la población adulta joven es la principal afectada por la enfermedad. Este grupo donde se incluyen los adultos jóvenes es el que mayor actividad ante la sociedad presenta, por lo que puede estar influenciado por un grupo de factores de riesgo como las preocupaciones ante la formación y conducción de la familia, el trabajo y las nuevas responsabilidades que se van adquiriendo en este período de la vida, que pudieran ser las causas que favorecen la aparición de la enfermedad al estar sometidos a estrés. 

De igual modo cuando se analizó la EAR dentro del total que padecen la enfermedad donde hay predominio del sexo femenino, lo cual es coincidente con estudios a nivel mundial ^17^^,^^18^ y en Cuba ^19^. Este predominio se debe a que incluso se ha observado presencia de aftas durante el ciclo menstrual y la menopausia, debido al aumento de progesterona y la disminución de estrógenos, lo que reduce la queratinización de la mucosa y aumenta la fragilidad capilar ^19^.

Al analizar los resultados y la clasificación clínica de la estomatitis aftosa recurrente se encontró un importante predominio de la aftosis menor sobre la aftosis mayor y herpetiforme. Los resultados obtenidos en la investigación coinciden con estudios realizados por Ansari en Iran ^21^, Gasmi en Francia ^18^ y Boza en Costa Rica ^7^, donde más de la tercera parte de los pacientes con EAR presentaban aftosis menor, muy superior al resto de las aftosis mayor y herpetiforme. En Cuba, los estudios demuestran igualmente un predominio de la aftosis menor, resultados que coinciden con los de esta investigación ^4^.

Este predominio de la aftosis menor sobre la aftosis mayor y la aftosis herpetiforme se presenta de forma mayoritaria, con una proporción aproximada de 1 de cada 10 pacientes. La mayor frecuencia relacionada con el tiempo de permanencia de la lesión correspondió entre 1 y 2 semanas, lo que coincide con autores a nivel mundial como Rodríguez, quien plantea que este tipo de lesiones permanecen en los pacientes pasados los 9 o 10 días ^22^.

De igual manera, otros estudios en Cuba plantean que la permanencia de la lesión por más de 7 días es más frecuente, resultados similares a los de esta investigación ^2^.

La demora de más de 7 a 10 días en cicatrizar las lesiones producto a una EAR puede estar relacionada con un trastorno en el proceso de cicatrización normal producto de la influencia de factores de riesgo como enfermedades asociadas que tienen entre sus consecuencias un retardo en la cicatrización, por ejemplo, la diabetes mellitus, u otros factores favorecedores de estas lesiones.

La localización más frecuente en la investigación fue a nivel de borde y punta de la lengua, fondo del surco vestibular, suelo de la boca y mucosa interna de los labios. Otros estudios, como los realizados por Rodríguez en México, dan como localizaciones más frecuentes la lengua, el suelo de la boca y la mucosa labial ^23^.

Otras investigaciones realizadas en Cuba, como la de Díaz ^(^^1^, encontraron como localizaciones más frecuentes los labios carrillos y la lengua. Aunque no siempre en los diferentes estudios la frecuencia aparece en el mismo orden, sí coinciden las zonas más frecuentemente lesionadas. Es posible que exista una relación entre las partes móviles de la boca y las lesiones producto a la estomatitis aftosa bucal.

Entre los factores que mayor incidencia tuvieron con la EAR y que fueron encontrados en la investigación tenemos las alteraciones psicosomáticas y las alteraciones gastrointestinales, y en menor grado otros factores. Estudios realizados por Ajmal ^24^ en Arabia Saudita muestran al estrés psicológico como un factor frecuente y principal en la aparición de EAR. Otras investigaciones como Qi ^25^ en China demostraron que es el resultado de la combinación de "múltiples factores" en el sistema digestivo, el sistema genético, el sistema inmunológico y el microambiente oral.

Karambelkar ^26^, en la India, encontró entre los factores de riesgo más frecuentes el estrés, el trauma bucal, la comida picante y la menstruación. Nuevamente, el estrés aparece como principal dentro de las posibles causas favorecedoras de la enfermedad. Todos estos resultados coinciden con los hallados en esta investigación.

Algunos autores en Cuba, como Díaz ^1^ y Bosch ^19^, refieren al estrés como el principal factor de riesgo; por otra parte, Leal ^9^ menciona entre los principales al estrés, las alteraciones inmunológicas y gastrointestinales, los cuadros alérgicos y los cambios hormonales.

Durante el periodo de la investigación, surgió en nuestro país la epidemia de COVID-19, enfermedad extremadamente contagiosa con alto riesgo para la vida, lo que generó que la población, en mayor o menor grado, se expusiera a una situación de estrés continuo, lo que favoreció la aparición de las lesiones representativas de la enfermedad aftosa recurrente.

Este tipo de estrés se sobreañade a las ya existentes situaciones de la vida cotidiana a las que está expuesta esta población en cualquiera de los grupos de edades estudiados, lo que, por supuesto, agrava este tipo de situación, relacionada con las alteraciones psicosomáticas.

Todos los pacientes que presentaron EAR mostraron, en mayor o menor medida, estas alteraciones. A esto se le agrega como factor secundario y relacionado también con el estrés las alteraciones gastrointestinales, aunque estas últimas también pueden ser consecuencias de otras causas, pero ambas influyen en la aparición de la EAR.

## CONCLUSIONES

La estomatitis aftosa recurrente tuvo una prevalencia de más del 30% de la población estudiada, con un predominio en el sexo femenino y los adultos jóvenes. La aftosis menor constituyó la lesión más frecuente en los pacientes junto a un tiempo de permanencia de más de 10 días. Las zonas de localización más frecuentes fueron la lengua, el fondo del surco vestibular, el suelo de la boca y la mucosa interna de los labios, con posible existencia de una relación entre las partes móviles de la boca. El estrés fue el principal factor de riesgo, agudizado por la presencia de la COVID-19.

## Financiamiento:

Ninguno
